# How to Naturally Support the Immune System in Inflammation—Essential Oils as Immune Boosters

**DOI:** 10.3390/biomedicines11092381

**Published:** 2023-08-25

**Authors:** Magdalena Grazul, Paweł Kwiatkowski, Kacper Hartman, Anna Kilanowicz, Monika Sienkiewicz

**Affiliations:** 1 Department of Pharmaceutical Microbiology and Microbiological Diagnostic, Medical University of Lodz, Muszynskiego 1, 90-151 Lodz, Poland; 2 Department of Diagnostic Immunology, Pomeranian Medical University in Szczecin, al. Powstancow Wlkp. 72, 70-111 Szczecin, Poland; 3 Department of Toxicology, Medical University of Lodz, Muszynskiego 1, 90-151 Lodz, Poland

**Keywords:** immunomodulation, inflammation, cytokines, chronic infections, secondary metabolites, essential oils

## Abstract

Efficient functionality of the immune system is needed to fight against the development of infectious diseases, including, among others, serious recurrent chronic infections. Research has shown that many modern common diseases, such as inflammatory bowel diseases and cardiovascular diseases, e.g., thromboembolism, cancer, obesity, or depression, are connected with inflammatory processes. Therefore, new, good stimulators of the immune system’s response are sought. They include synthetic compounds as well as biological preparations such as lipopolysaccharides, enzymes, bacterial metabolites, and secondary metabolites of plants, demonstrating a multidirectional effect. Essential oils are characterized by many invaluable activities, including antimicrobial, antioxidant, anti-inflammatory, and immunostimulating. Essential oils may stimulate the immune system via the utilization of their constituents, such as antibodies, cytokines, and dendritic cells. Some essential oils may stimulate the proliferation of immune-competent cells, including polymorphonuclear leukocytes, macrophages, dendritic cells, natural killer cells, and B and T lymphocytes. This review is focused on the ability of essential oils to affect the immune system. It is also possible that essential oil components positively interact with recommended anti-inflammatory and antimicrobial drugs. Thus, there is a need to explore possible synergies between essential oils and their active ingredients for medical use.

## 1. Introduction

Proper functioning of the immune system is a key factor in the prevention and treatment of many different diseases, e.g., infections and cancers. Adverse effects of antibiotics and their bacterial resistance have prompted researchers to attempt to influence the host immune response to an infection using compounds demonstrating immunomodulatory properties, called biological response modifiers (BRM). These substances may affect certain immune mechanisms either as immunostimulators or immunosuppressants [1].

Nowadays, most available substances that might affect the human immune system are of natural origin. For instance, in lipopolysaccharide (LPS), many different enzymes and metabolites are isolated from bacteria, while secondary metabolites, commonly used in the production of immunoboosters, are mostly extracted from plants. Also, products made by animals, e.g., reptile venom, may be utilized. In recent years, compounds derived from medicinal plants have appeared to be highly interesting immunomodulators [1]. Essential oils, characterized by a wide spectrum of health-supporting activity, including antimicrobial, anticancer, anti-inflammatory, and immunomodulatory properties, make up a particularly interesting group of herbal metabolites.

## 2. Action of the Human Immune System

The principal role of the immune system is to protect the host from outside distress, e.g., pathogens. Its activity comprises two variants of systems: innate and adaptive immunity [2]. The innate immune system acts as the first line of response and is responsible for an adequate reaction to injury. Its rapidity is a key factor in the elimination of the transmission of pathogens through their recognition, internalization into phagocytes, and digestion. This process engages receptors called pattern recognition receptors (PRRs), responsible for the recognition of specific structures of microorganisms, pathogen-associated molecular patterns (PAMPs), and damage-associated molecular patterns (DAMPs) [3]. During the next step, several types of cytokines, e.g., interleukin 1 (IL-1), IL-6, IL-8, and tumor necrosis factor (TNF), stimulate neighboring cells [4] to secrete chemokines to begin the inflammatory response [5]. Also, activation of lymphocytes may be related to inflammatory processes [5,6]. IL-6, which plays a role in the differentiation of B cells, stimulation of immunoglobulin production, induction of the acute phase response, and stimulation of inflammatory processes, is one of the most important cytokines involved in the host defense system as it demonstrates a broad spectrum of immune and hematopoietic properties. Also, IL-8 (a chemokine called C-X-C motif ligand 8; CXCL8) is involved in the immunological response via induction of chemotaxis in target cells and stimulation of phagocytosis [5,7]. TNF (the inflammatory cytokine) is generated by macrophages or monocytes during acute inflammation processes that lead to necrosis or apoptosis [8]. It is also involved in leukocyte adhesion, the control of blood coagulation, and the indirect induction of fever [9,10]. 

Secretion of inflammatory mediators takes place via proteins called Toll-like receptors (TLRs) and leads to the maturation of dendritic cells and phagocytes involved in tissue homeostasis, which may prime the adaptive immune response during the next step by inducing T cell maturation and T helper type 1 (Th1) polarization [11,12].

The adaptive immune system provides a delayed but definitely more specific response. It generates and retains memory and comprises humoral and cell-mediated responses, in which two types of cells are involved: T and B cells. Antigen-presenting cells stimulate these cells upon recognizing either free antigen or bound antigen. Activated B cells produce antibodies, which are responsible for, e.g., toxin inactivation, bacterial opsonization, pathogen fagocytosis, and activation of the complement system. Five classes of antibodies are produced: IgM, IgG, IgE, IgA, and IgD. For instance, IgM and IgG immunoglobulins, which are present in the blood serum, are involved in recognizing and binding to specific antigens–pathogens and toxins, hereby taking part in their destruction or elimination [13]. They may also play a role as antigen-presenting cells, e.g., for a primed specific T cell, or be involved in cytokine production [14], while T cells produce cytokines responsible for B cell division and differentiation into a mature, antibody-producing plasma cell [15]. 

## 3. Influence of the Immune System on Wound Healing

According to professional literature, 1 in 10 diabetic patients worldwide is affected by major complications related to chronic non-healing wounds [16]. Chronic non-healing wounds, e.g., those that affect diabetic patients, increase the risk of infection [17]. 

Our skin is protected from unwanted external conditions, e.g., microbes, chemicals, and injuries. Inflammation of the wound is a host response to microorganisms that damage viable host tissue, thereby affecting the wound healing process. Inflammation is supposed to eliminate or at least reduce unwanted changes made by an injurious agent. The innate and adaptive immune systems are important regulators of processes involved in wound healing through the promotion of cellular cross-talk and the secretion of signaling molecules, e.g., cytokines, chemokines, and growth factors. It has to be noted that epithelium not only serves as the body’s first-line mechanical barrier but is also a part of innate immunity processes, e.g., being a regulator of inflammation via expression of chemotactic agents of human cathelicidin LL-37 in keratinocytes during skin inflammation and being a promoter of resistance to skin infections by the production of antimicrobial peptides (AMPs) [18,19]. The relationship between epithelial regeneration and AMP production has been proven. Some types of AMPs promote wound-edge keratinocyte proliferation and migration [20]. Additionally, keratinocytes express proteins called TLRs, which are involved in the production of cytokines and chemokines [5]. There are many different types of TLRs expressed throughout the normal human epidermis, but TLR4 is specifically needed in early skin wound healing [21]. TLR activation may affect wound healing in a dose-dependent manner, as it may suppress growth at higher concentrations but promote regeneration at lower concentrations [22]. 

Various immune cells take part in the maintenance of homeostasis in healthy skin. Dendritic cells, macrophages, T cells, and neutrophils are all involved in wound healing [16]. In acute infections, interleukins are secreted into blood vessels from local tissue sites [7]. It is well documented that T cells may affect the wound environment in different ways as they secrete several types of cytokines. T lymphocytes play a role in the production of growth factors and as immunological effector cells during wound healing [23]. Both innate and adaptive responses are needed for cutaneous immunosurveillance and wound healing [15,24]. Acute wound healing includes the production of specific signaling molecules, cytokines, chemokines, and growth factors. It is noteworthy that overproduction of cytokines and proteases results in persistent inflammation, thus affecting wound healing. Therefore, the immune processes must be monitored to ensure successful wound healing [24]. 

Neutrophils, which belong to the innate immune response, play a role in both acute and hard-to-heal wound inflammation. In acute infections, they lead to the phagocytosis of invading microorganisms by the secretion of extracellular traps (NETs), which capture and inactivate bacteria [25]. The situation becomes more complicated when microbes are able to produce biofilm. It has been proven that a chronic biofilm infection is related to a permanent and lower-grade host inflammatory response (lower levels of IL-1β and TNF expression) when compared with an infection of acute wounds [26]. Additionally, Moser et al. proved that the persistent inflammatory response may lead to host tissue damage, fibroblast senescence, and inhibited secretion of growth factors required for wound healing if the innate host immune system is not able to eradicate biofilm. Therefore, fighting wound biofilm, which occurs during chronic infection, involves an additional step in the immune response. At the beginning of an infection, neutrophils are recruited similarly as in the event of an acute infection, but in the process of biofilm maturation, pro-inflammatory cytokines, e.g., interleukins and TNF, are produced. This step takes place when the host’s response becomes overwhelmed. It prevents continuous oxidative stress, supports the production of growth factors, and generally controls infection [27,28]. 

## 4. Influence of Selected Essential Oils on the Immune System

According to the World Health Organization (WHO, Geneva, Switzerland), over 60% of the world’s population confirms the therapeutic effect of folk medicine [29]. Many different scientific articles indicate that herbs used in traditional medicine contain various compounds demonstrating biological and therapeutic activities [30]. Many plants produce essential oils when subjected to stressful conditions. Essential oils have a wide range of health promoting properties, and they are also highly safe due to their low toxicity and minor side effects. 

Essential oils are complex mixtures extracted from different parts of plants (stem, flowers, or leaves) through steam distillation or the use of specific solvents that have been used in traditional medicine for ages [31]. The most known application of essential oils, i.e., aromatherapy, may successfully improve the quality of life of patients suffering from serious diseases, such as cancer, as it reduces anxiety and pain [32,33]. 

The biological activity of essential oils is mainly a result of the activity of their constituents through synergistic or antagonistic effects [34]. Essential oils may also stimulate the immune system and thus become an alternative to conventional treatment of many different diseases and other health problems, e.g., cancer, allergies, wounds, and inflammatory diseases [35], via the utilization of constituents of the immune system, such as antibodies, cytokines, and dendritic cells [36,37]. Some essential oils may stimulate the proliferation of immune-competent cells, including polymorphonuclear leukocytes, macrophages, dendritic cells, natural killer cells, and B and T lymphocytes [38,39]. For instance, constituents of essential oils produced by *Eucalyptus* stimulate phagocytosis by macrophages [40], while *Nigella sativa* essential oil stops the proliferation of CD4+ and CD8+ lymphocytes in vitro [41]. 

Therefore, together with their constituents, they are considered promising therapeutic agents, which could be an alternative/support to currently used antibiotics and other drugs [42]. According to professional literature reports, essential oils may display similar or even greater immunomodulatory properties compared to conventional drugs [43,44]. Yet it has to be kept in mind that essential oils, if used improperly, might have some side effects. However, they are not cytotoxic if used at low concentrations [39,45].

**Figure 1:**
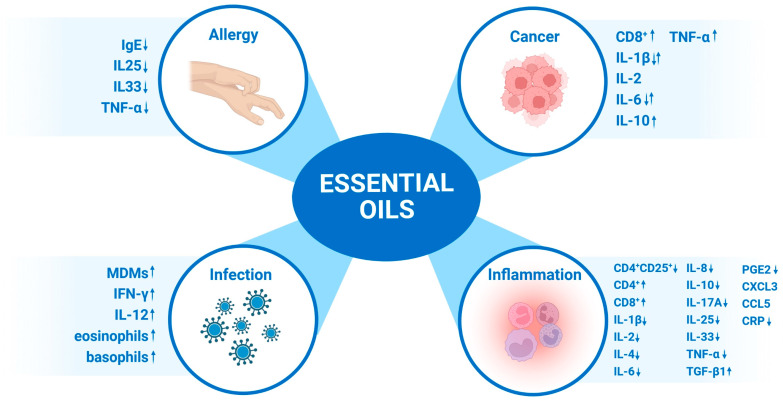
Immunomodulatory and anti-inflammatory properties of essential oils. Created with Biorender.com (↑ stimulation, ↓ reduction).

This review summarizes a literature-based search focused on the abilities of essential oils to affect the immune system. By searching the keywords “immune system and essential oil”, “immune response and essential oil”, “essential oil and inflammation”, and “essential oil and wound” in PubMed, Web of Science, and Science Direct, we screened out articles with high correlation based on the influence of essential oils on the human immune system and their potential application as immunomodulators of this system. We decided to exclude studies focused on the influence of Eos on animal immune systems for veterinary purposes. Due to the fact that a very good review related to the application of essential oils in immunology was published in 2019 [46] and a lot of new interesting data related to this subject were also published, we decided to analyze the research reports that have been published within the last 5 years. Here, we would like to present an overview of the biological properties of essential oils produced by plants that we regard as interesting, giving special consideration to their immunomodulatory and anti-inflammatory properties (Figure 1). The detailed composition of all essential oils discussed in this manuscript and a summary of their influence on the immune system (activity) are presented in Table 1 at the end of the section. The influence of the most common essential oil constituents on the immune system (activity) and their chemical classification are presented in Table 2 at the end of the section.

The wild plant *Myrtus communis* (*M. communis*, myrtle) is known in traditional medicine as a remedy for stomach aches, wound healing, hemorrhoids, etc. [47], as its leaves, fruits, roots, and berries display, for example, anti-inflammatory, analgesic, antioxidant, anticancer, anti-diabetic, anti-mutagenic, and neuro-protective activity [48]. It releases essential oils whose insecticidal, antioxidant, anti-inflammatory, antibacterial (against *Staphylococcus aureus*, *Listeria monocytogenes*, *Pseudomonas aeruginosa*, *Escherichia coli*, *Klebsiella pneumoniae*, etc.), antiviral (against Herpes simplex), and antifungal (against *Candida* spp., etc.) properties are widely used in traditional herbal medicine. These essential oils are rich in terpenes, terpenoids, and phenylpropanoids [49]. Shaapan et al. revealed the prophylactic effects of *M. communis* essential oils against chronic toxoplasmosis induced by the Tehran strain of *Toxoplasma gondii* in mice. According to the literature, toxoplasmosis is controlled by cellular immunity, so the authors evaluated the effect of the tested myrtus oil on chosen innate immunity mediators such as interferon gamma (IFN-γ) and IL-12 [50]. IL-12 and IFN-γ. All of these are involved in the control of the synthesis of nitric oxide, which is the initial effector in the immune system response against toxoplasmosis [51]. The studies showed that the IFN-γ and IL-12 mRNA levels were significantly higher in mice treated with essential oils when compared to the control groups. Mahmoudvand et al. proved that the mentioned essential oil is not significantly toxic at doses up to 0.4 mL/kg to BALB/c mice when administered orally for 14 days [52]. However, further, more advanced studies on the choice of optimal concentrations, the most active fraction or extracts, the mechanism of action, and pharmacokinetics are still needed [53,54,55].

Shin et al. explored the immunomodulatory effects of essential oils extracted from *Chamaecyparis obtusa* on house dust mite-induced mucosal inflammation. *Chamaecyparis obtusa* (*C. obtuse*, Cupressaceae) is a species of cypress commonly found in the southern part of Korea and central Japan that produces essential oils containing, e.g., monoterpenes, sesquiterpene hydrocarbons, and oxygenated sesquiterpenes. It also possesses antibacterial, antiatopic, and anti-inflammatory properties [56]. The mentioned essential oil significantly suppresses the secretion of IL-25 and IL-33 from epithelial cells (critical regulators of Th2 immune responses), which may occur as a response to allergens related to the development of airway allergic inflammation, in a dose-dependent manner [57,58]. It also controls prostaglandins (PGs) and TNF gene expression, thus blocking LPS-induced inflammation as well as the expression and activity of nuclear-kappa B factor (NF-κB) and activator protein 1 (AP-1), leading to inhibited production of IL-6 and IL-8 from bronchial epithelial cells [48]. Thus, it may affect innate and adaptive immune responses involved in the inflammation process. It is noteworthy that a concentration of essential oil lower than 0.1% did not affect the viability of nasal epithelial cells treated with allergens related to the development of airway allergic inflammation, and according to some authors, plain oil extracted from C. obtusa was approximately 10 times more toxic to nasal epithelial cells than its microencapsulated form [59].

*Nigella sativa* (*N. sativa*, Ranunculaceae) produces seeds known as black cumin, Haba al-Barakah (blessed seeds), or miracle herb. The seeds were called “herbs from heaven” by ancient herbalists [60,61]. Its beneficial properties have been used widely all over the world in traditional medicine, for example, in Unani, Ayurveda, Tibb, and Siddha cultures for a long time in therapies for many different diseases such as asthma, bronchitis, rheumatism, headache, back pain, paralysis, inflammation, and hypertension [62]. It contains many different compounds that are able to inhibit chronic inflammation and stimulate a healthy immune response. Additionally, they may affect autophagy dysfunction, oxidative stress, ischemia, inflammation, and bacterial and viral infections. Moreover, topical application of this essential oil showed an antiseptic and local anesthetic effect, as well as helping to get rid of skin blisters and eczema [63]. Its main constituents are nigellone, thymoquinone, thymohydroquinone, dithymoquinone, thymol, carvacrol, α- and β-pinene, d-limonene, d-citronellol, p-cymene, carvacrol, trans-anethole, 4-terpineol, longifolene, and α-hederin [61,64]. The most biologically active element is thymoquinone. According to literature reports, it is responsible for the majority of the above-mentioned properties of cumin essential oil, and its antioxidant, anticancer, and antibacterial properties were confirmed in numerous studies [65,66,67,68,69,70,71,72]. Several studies on animal models proved that the essential oil isolated from black cumin also exhibits good anti-inflammatory activity related to the presence of thymoquinone [73]. Hossen et al. proved that it affects the activity of pro-inflammatory factors (TNF, IL-6, and IL-1β) in LPS-stimulated murine macrophage-like RAW264.7 cells [74]. Additionally, Ebrahimi et al. proved that α-hederin administered at a concentration of 0.02 mg/kg in ovalbumin (OVA)-sensitized rats, as in the asthma model, has an influence on IL-2 and IL-17 secretion pathways, changing miRNA-133a expression [71,75]. It should be pointed out that freshly extracted essential oil reduced the IL-6 level in human pre-adipocytes with low-grade inflammation, while stored oil decreased the IL-1β level [76]. At a concentration of 400 mg/kg, it also changed the level of pro-inflammatory cytokines (IL-6, IL-12, and TNF) in paw exudates and sera in rats with carrageenan-induced paw edema [77]. Moreover, external application of a balm stick containing 10% essential oil in rats with paw edema inhibits edema (60.64%), reduces leukocyte count (43.55% lower than control), and lowers TNF level (50% lower than control), thereby significantly reducing acute and sub-acute inflammation at the inflammation site [73]. Its ability to suppress inflammation is related to the activation of anti-inflammatory signaling pathways (NF-κB and TLR signaling). It can stimulate immunity via one of several pathways: 1. modulation of innate as well as adaptive immune components; 2. prevention of apoptosis by upregulation of pro-survival signals and downregulation of pro-apoptotic signals (PI3K/Akt, JNK, and mTOR); 3. induction of autophagy (silent mating type information regulation 2 homologue 1 [SIRT1] signaling); 4. priming of energy metabolism (AMPK-SIRT1-PGC-1α and PPARγ signaling); 5. stimulation of the growth factor pathway (PI3K/Akt signaling); 6. upregulation of lipoprotein receptor-related protein 1 (LRP1), which leads to protein clearance [78]. 

Moreover, it has been documented that cumin essential oil reduces the inflammation process in LPS/IFN-γ or H_2_O_2_-activated BV-2 microglial cells by reducing the level of cytokine (IL-2, IL-4, IL-6, IL-10, and IL-17a) and chemokine (CXCL3 and C-C motif ligand 5 [CCL5]) expression, thus protecting against neuroinflammation related to Alzheimer’s disease [79]. It may also protect from neuroinflammation by inducing autophagy via activation and nuclear accumulation of SIRT1 in LPS-activated BV-2 microglia [80]. Moreover, by downregulating the expression of TLR signaling components and their downstream NF-κB effectors and interferon regulatory factor 3 (IRF-3), cumin essential oil increases inflammation processes in the Alzheimer’s disease model [81]. 

Also, a few preclinical and clinical studies revealed the immunomodulatory properties of black cumin. For instance, Sheik et al. proved that an ethanolic extract of black cumin increases the macrophage population of the murine cell line J774A.1 [82] and supports the phagocytic properties of several different macrophage populations [83]. Koshak et al. confirmed that the essential oil extract supports the immune response by reducing IL-2, IL-6, and prostaglandin E2 (PGE2) in primary T-lymphocytes and IL-6 and PGE2 in primary monocytes, which are asthma-related inflammatory mediators [84]. It can also activate macrophages and sustain the total number of leukocytes, neutrophils, eosinophils, basophils, lymphocytes, and monocytes in Salmonella Typhimurium-infected rats [85]. 

Interestingly, black cumin and its constituents improve the anticancer activity of other recommended chemotherapeutic and natural compounds that are able to prevent cancer. For instance, when applied together with 5-fluorouracil at a concentration of 35 mg/kg/day, p.o., 3 days/week for 9 weeks, they improved its cytotoxicity in AOM-instigated colon cancer rats by reducing NF-κB expression [86]. An application of black cumin at a concentration of 10 mg/kg/day; i.p. for 14 days in combination with piperine significantly diminishes tumor size and leads to apoptosis via downregulation of vascular endothelial growth factor (VEGF) and improvement of IFN-γ and IL-2 levels [78,87].

*Houttuynia cordata* Thunb. (*H. cordata*; Saururaceae) is a widely grown plant in Asia and commonly used in folk medicine due to its biological activity. For instance, in China, it has been used as a remedy for pneumonia and lung abscesses for ages. It might be applied in combination with other medicines against dysentery, colds, fevers, and mumps. Nowadays, it is still eaten and applied in traditional medicine, for instance, in the Yarlung Zangbo Valley in Assam, India [88]. Its wide spectrum of biological activity is attributed to the presence of flavonoids and essential oils. The main constituents of the essential oil are monoterpenes, sesquiterpenes and their oxides, oxidized diterpenes and phenylpropene derivatives, nonyl ketones (2.10–40.36%), bornyl acetate (0.4–8.61%), and β-myrcene (2.58–18.47%), but their composition differs between the aboveground and underground parts of the plant [89,90]. Aboveground parts, rather than underground elements, contain a concentration of 2-undecanone, myrcene, ethyl decanoate, ethyl dodecanoate, 2-tridecanone, and decanal. Differences observed in the antibacterial activity of essential oils isolated from chosen parts of *H. cordata* by some researchers relate to the composition of particular parts of this plant [91]. According to literature reports, the plant is able to decrease the secretion of inflammatory factors, thereby moderating lung injury and rheumatoid arthritis. In addition, it may strengthen the immune barriers of the vagina, oral cavity, and intestinal tract against pathogen infection. It is able to alleviate the pathological influence of bleomycin on lung tissue [92] by upregulating IFN-γ levels and inhibiting the transforming growth factor β1 (TGF-β1)/Smad signaling pathway in mice [93,94]. 

*Thymus vulgaris* L. (*T. vulgaris*) is another herb that produces biologically active essential oil. According to professional literature, it possesses a wide spectrum of properties, e.g., antioxidant, antibacterial, antispasmodic, anthelmintic, immunomodulatory, anti-inflammatory, and antiseptic [43]. These activities are attributed to its constituents, such as thymol and eugenol, which are phenolic compounds. It must also be taken into account that the amount of constituents and especially active ingredients in *T. vulgaris* oil may vary depending on environmental conditions, plant harvesting seasons, and the geographical area [43]. The anti-inflammatory activity of essential oil extracted from *T. vulgaris* is probably related to its capability to diminish the production of proinflammatory cytokines [95]. Ocaña et al. proved that *T. vulgaris* EO is able to elevate inflammation via reduced secretion of inflammatory mediators such as TNF, IL-1β, C-reactive protein (CRP), and IL-8, as well as an increase in anti-inflammatory mediators such as IL-10 production processes [96]. Major constituents of essential oils involved in this process seem to be thymol, which affects the properties of an inflammatory process marker called elastase, produced by activated neutrophils, and inhibits cyclooxygenase (COX), as well as p-cymene, which inhibits NF-κB and mitogen-activated protein kinase (MAPK) signaling pathways and affects TNF and IL-1β secretion [97,98]. It is also noteworthy that thymol and carvacrol are able to suppress the activity of transcription factors responsible for the initiation of cytokine expression in T cells, including AP-1 and nuclear factors of activated T cells (NFAT). It is also able to arrest the production of IL-2 and IFN-γ, thus affecting T cell activation [99]. Ismail et al. observed that application of essential oil from T. vulgaris to animals affected by *E. coli* O157:H7 significantly diminished serum IL-6, IL-8, and TNF concentrations when compared to the control group [8,100]. As literature reports show, thyme essential oil may also affect the synthesis of LPS-mediated nitric oxide and IFN-γ [8,101]. This observation is also worth stressing.

Also, *Artemisia judaica* L. (*A. judaica*), a perennial plant from the Asteraceae (Compositae) family, is commonly used due to its pro-health properties [8]. It commonly grows in the Mediterranean region, including Algeria, Libya, Egypt, Jordan, and Saudi Arabia [10,102,103]. It is famous for its anticancer, antimicrobial, antiviral, antidiabetic, antioxidant, hepatoprotective, and anti-inflammatory activities. Its application to hyperglycemia, heart diseases, inflammatory disorders, and arthritis is known in traditional medicine [102,104]. Moreover, it is able to improve vision and the immune system [105,106]. It produces essential oils, which contain, e.g., monoterpenes and cinnamic acid derivatives, known for their excellent antioxidant properties in vitro and in vivo. Hamdoon et al., among other authors, noted that they can reduce TNF and promote pro-angiogenic/anti-inflammatory TGF-β1, which is involved in wound healing, angiogenesis, immune regulation, and cancer. However, it has to be pointed out that an application of this essential oil did not affect IL-1b and IL-6, which help TGF-β1 in Th17 differentiation, aggravating inflammation. Additionally, the level of IL-10, a regulator of the immune response that limits the inflammatory process, was also promoted in wound healing tissue when essential oil was applied [9,107,108]. 

The anti-inflammatory as well as antioxidant activity of *A. judaica* essential oil is related to the presence of oxygenated monoterpenes [9] known for their involvement in wound healing: thujone, 1,8-cineole, camphor, borneol, and methyl cinnamate. Moreover, 1,8-cineole suppresses the inflammatory markers TNF, IL-6, IL-8, leukotriene B4 (LTB4), PGE 2, and IL-1; it also downregulates 5-lipoxygenase (LOX) and COX [10,109,110].

Patchouli (*Pogostemon cablin*, *P. cablin*) essential oil has been known for its healing properties since ancient Egyptian times. It is noteworthy that it is able to differ M1 to M2 macrophage phenotypes and change the inflammatory milieu of ApcMin/+ mice, inhibit CD4+CD25+, and stimulate CD4+ and CD8+ cells in the spleen, blood, mesenteric lymph nodes (MLNs), and Peyer’s patches (PPs) of treated mice [111].

*Echinacea purpurea* (*E. purpurea*) L. Moench (Asteraceae) is widely known in the United States, Canada, and Europe for its medicinal properties. *E. purpurea*, *E. angustifolia,* and *E. pallida* are the most known biologically active species from the *Echinacea* genus [112]. The main elements of the essential oil extracted from Echinacea are terpenes, such as germacrene D, polyacetylenes, highly unsaturated alkamides, and phenolic compounds [113,114]. According to literature reports, it helps in wound healing, supports the immune system, and diminishes respiratory problems caused by bacterial infections. It is postulated that its antimicrobial and immunomodulatory properties may be linked to each other due to synergistic effects on the host immune system. Echinacea may affect the immune system by stimulating monocytes and natural killer cells [112,114,115].

Cardamom (Elettaria cardamomum Maton, Zingiberaceae) is a perennial herb commonly used as a spice in traditional Indian cuisine. It is called “the Queen of Spices”, as it is listed as the third most precious spice after saffron and vanilla [116]. Its essential oils are known in folk medicine and are applied in the treatment of colds, bronchitis, and asthma [117]. The anti-oxidant, anti-inflammatory, anti-microbial, and anti-cancerogenic properties of essential oils result from the biological activity of phenolic compounds [118,119,120].

An immune-competent patient affected by *Camphylobacter* spp. requires only symptomatic treatment and fluid and electrolyte replenishment, whereas an immune-compromised patient should usually be administered antibiotic treatment with macrolides or fluoroquinolones. Additionally, in some rare cases, post-infectious autoimmune diseases like chronic inflammatory bowel diseases may occur [121]. 

Heimesaat noted that application of cardamom essential oil to mice suffering from acute *C. jejuni*-induced enterocolitis (*C. jejuni*-infected microbiota-depleted IL-10^−^/^−^ mice) may induce an anti-pathogenic and immune-modulatory effect by reduction of pro-inflammatory mediator secretion in the intestinal tract, extra-intestinal, and systemic compartments and by general improvement of clinical outcome during acute campylobacteriosis. These beneficial effects may be observed at least one day following the commencement of the treatment (i.e., day 3 p.i.). However, it should be kept in mind that these results might be, at least partly, related to the antimicrobial activity of applied cardamom [122,123,124]. A diminishing pro-inflammatory immune response during *C. jejuni* infection in IL-10^−^/^−^ mice is related to a reduced number of macrophages, monocytes, and T lymphocytes in the colonic mucosa and lamina propria and a lower secretion of nitric oxide, TNF, and IFN-γ in the intestinal tract. Additionally, in vitro studies showed that cardamom suppresses the secretion of Th1 cytokines, including IFN-γ [125], in murine splenocytes, which also appeared to be true for nitric oxide and TNF production by mouse peritoneal macrophages [125]. It is noteworthy that suppression of inflammatory processes in alveolar macrophages in vitro is related to the activity of 1,8-cineole, i.e., the main constituent of essential oil [124,126].

Also, essential oil extracted from the peel of the Citrus sudachi (sudachi oil, Rutaceae) plant is able to suppress antigen-induced lymphocyte activation as well as the properties of antigen-presentation-related molecules on dendritic cells in vitro. Sudachi oil also suppresses antigen-specific T cell induction in vivo in mice immunized with OVA [127].

*Euodia ruticarpa* (*E. ruticarpia*) (Rutaceae), also known as Tetradium ruticarpum, but especially its fruit, called Tetradii fructus, Evodiae fructus, or Wu Zhu Yu, is commonly applied in traditional as well as clinical medicine in East Asia, e.g., in Korea, Japan, and China, due to a great variety of health benefits, e.g., anti-inflammatory and anti-cancer properties. It has been used to treat post-eating nausea, vomiting, diarrhea, abdominal pain, headache, pelvic inflammation, or bacterial infections [32,128,129]. According to Yeh et al., the main constituents of steam-distilled essential oil, such as 2-(3-methoxyphenyl)-4H-1-benzopyran-4-one, N,N′-diphenyl-1,4-benzenediamine, benzoic acid, N-propylbenzamide, and dipropylene glycol dibenzoate, are responsible for *E. ruticarpa’s* anti-cancer properties. They are able to inhibit the PC-3 prostate cancer cell line by regulating Th1 (IL-2) and Th2 (IL-10) cytokine production by splenocytes (the spleen is strictly involved in immunological processes in the human body) [130]. Furthermore, the levels of IL-1β, TNF, IL-6, and IL-10 secreted by macrophages increased. Considering the fact that synergistic interactions of IL-2 and IL-10 cytokines advance the CD8+ T cell cytotoxic effect, the authors concluded that the essential oil is able to support anti-cancer immunity. It may also activate natural killer (NK) cells and B cells, generating even better anti-cancer results [131,132,133]. According to Tahvildari et al., IL-2 treatment seems to be a promising direction to combat both autoimmune and inflammatory diseases. Moreover, it has been approved by the US Food and Drug Administration for the treatment of cancer. The PC-3 cell line is hormonally independent, so traditional hormonal therapy cannot be applied. In this case, an alternative therapy that fights cancer indirectly via immune cells might be invaluable [32,134,135]. 

The Eucalyptus plant (Myrtaceae), used by Australian Aboriginals in the treatment of inflammation and wound infections, is one of the most known herbs that is able to produce essential oil [136]. Nowadays, it is also used as a support in the therapy of infectious and respiratory diseases [137,138]. Its main constituent, 1,8-cineole (also known as eucalyptol), as well as terpenes such α-pinene and limonene, are characterized by a wide spectrum of biological activities [139,140]. For instance, its topical application inhibits edema and enhances vascular permeability in IgE-mediated allergic dermatitis. It may also stimulate the immune system, e.g., the immune function of the respiratory tract. It can also be used as an adjuvant in some infections and inflammatory diseases. This essential oil is able to stimulate monocyte-derived macrophage (MDM) activity in vitro [40,141,142]. According to Zonfrillo et al., it seems to improve the digestion process of microbial agents, e.g., the phagocytosis of zymosan particles by macrophages. Hence, it might be used as an adjuvant in the treatment of infectious and immunosuppressive diseases. It is noteworthy that pathogen internalization in this process takes place via stimulation of complement receptor (CR)-mediated phagocytosis soon after exposure to foreign microorganisms, which means that essential oils may affect the innate immune system already at the early stages of pathogen infection. It is known that this property of essential oils is attributed to the presence of eucalyptol. Nevertheless, further studies on this process are needed. The mentioned essential oil might also increase podosome density as well as augment colocalization at the podosomes of the actin core with the ring component vinculin, thereby taking part in macrophage motility and chemotaxis processes. Moreover, in the initial stages of treatment, this essential oil is able to significantly stimulate expression levels of the basic structural elements of the podosome ring: αM (CD11b) and β2 (CD18) integrins, involved in control of the relation between cell and extracellular matrix in processes related to cell movement, and form together the heterodimeric complex CD11b/CD18 (or CR3) [2].

**Table 1:** The influence of essential oils on the immune system and their composition.

Lp.	Plant Species	Activity	Main Constituents (>1%) (The Composition of Essential Oil May Differ Depending on e.g., a Part of the Plant, Geographical Area, Season When It Was Harvested) (–Means Lack of the Peesence of the Component in Tested Material)	Literature Source
1.	*Myrtus communis* (*M. communis*, myrtle)	Increases levels of IL-12 and IFN-γ mRNA in response to toxoplasmosis.	The composition of essential oil isolated from leafs of the plant grown in Jericho/Jenin/Tunisia: α-Pinene 3.95/10.22/15.59 o-Cymene 0.17/1.34/- 1,8-Cineole 24.32/31.55/16.55 Linalool –/21.65/13.30 cis-4-Thujanol 27.37/– α-Terpineol –/4.35/2.88 Myrtenol –/20/- Myrtenal 6.78/21/- Trans-4-Thujanol acetate 11.26/22/- Linalyl acetate 1.04/0.42/3.67 Myrtenyl acetate 0.62/2.89/20.75 α-Terpinyl acetate 1.77/1.30/- a-humulene -/-/1.29 limonene -/-/8.94 geranyl acetate -/-/2.99 methyleugenol -/-/1.17	[50,51,52,143,144]
2.	*Chamaecyparis obtusa* (*C. obtuse*, Cupressaceae)	It is able to inhibit inflammatory processes by: suppressing secretion of IL-25, IL-33 from epithelial cells, affecting PGs and TNF gene expression affects activity of NF-κB and AP-1 inhibiting production of IL-6, IL-8 from bronchial epithelial cells [48].	The composition of essential oils isolated from leafs of the tree grown in South Korea/from fruit of the tree grown in Sudan: α-Pinene 1.85/69.1 sabinene 10.97/11.6 myrcene 3.76/1.1 limonene 6.89/- γ-terpinene 3.13/- terpinene-4-ol 4.11/- isoBornyl acetate 8.85/- α-terpinyl acetate 13.71/- cadinene 1.57/- Ceder acetate 4.25/- epi-Bicyclosesqui-phellandrene 4.06/- β-Cubebene 1.12/- δ-Cadinene 2.95/- α-Elemene 2.32/- α-Cedrene 1.10/- Elemol 4.62/- Cedrol 1.75/- α-Selinene 1.34 /- β-Eudesmol 1.58/- Longifolene 2.03/- β-pinene -/3.1 δ-3-Carene -/12.1	[48,57,58,145,146]
3.	*Nigella sativa* (*N. sativa*, Ranunculaceae)	Inhibits inflammation by: activating NF-κB and TLR signaling pathway preventing apoptosis by upregulation of pro-survival signals and downregulation of pro-apoptotic signals (PI3K/Akt, JNK, and mTOR) inducing autophagy (silent mating type information regulation 2 homologue 1 [SIRT1] signaling) primes energy metabolism (AMPK-SIRT1-PGC-1α and PPARγ signaling) stimulating PI3K/Akt signaling pathway upregulates lipoprotein receptor-related protein 1 (LRP1) affecting IL-2 and IL-17 secretion pathways, changing miRNA-133a expression in the asthma model in rats affecting activity of TNF, IL-6, IL-1β in LPS-stimulated murine macrophage-like RAW264.7 cells reducing IL-2, IL-6, and PGE2 in primary T-lymphocytes and IL-6 and PGE2 in primary monocytes, which are asthma-related inflammatory mediators changing IL-6, IL-12, TNF levels and leukocytes count in rats with paw edema reducing IL-2, IL-4, IL-6, IL-10, and IL-17a levels, as well as chemokines (CXCL3 and C-C motif ligand 5 [CCL5]) expression in the Alzheimer’s disease model Freshly extracted oil reduces the IL-6 level in human pre-adipocyte, while stored oil decreases IL-1β level in rats with paw edema May increase proinflamatory processess in the Alzheimer’s disease model by: downregulating the expression of TLRs signaling components and their downstream effectors NF-κB and interferon regulatory factor 3 (IRF-3) Plays a role in combating infections by: sustaining the total number of leukocytes, neutrophils, eosinophils, basophils, lymphocytes, monocytes in Salmonella Typhimurium infected rats Plays a role in anticancer treatment because: in combination with piperine, it affects tumor size downregulates VEGF, improves IFN and IL-2 levels leading to apoptosis	The composition of essential oil of Iranian/Brazilian black cumin seed: α-Thujene 9.8/2.4 α-Pinene 3.1/1.2 Sabinene 2.2/1.4 β-Pinene 3.4/1.3 para-Cymene 37.3/- γ-Terpinene 2.0/- Linalool 9.9/- 4-Terpineol 1.0/- alpha-Terpineol 2.2/- Thymoquinone 13.7/11.8 Carvacrol 1.6/3.7 alpha-Longipinene 2.1/- Longifolene 6.4/5.7 Nerol -/1.3 Estragole -/1.9 Carvone -/2.0 Anisaldehyde -/1.7 Trans-Anethole -/27.1 p-Cymene -/9.0 Limonene -/4.3 Uvidine -/1.3 2(1H)-Naphthalenone -/2.6 Myristicin -/1.4 Fenchone -/0.1 Apiole -/1.0	[71,74,75,76,77,78,79,80,81,147,148]
4.	*Houttuynia cordata* Thunb. (*H. cordata*; Saururaceae)	May reduce side effects of bleomycine in cancer treatment as: upregulates the level of IFN-γ inhibits β1 (TGF-β1)/Smad signaling pathway in mice	The composition of essential oil isolated from dried aerial part of leafs/fresh whole grass of the plant grown in China: 1-Nonanol 3.10/0.49 Decanal 2.68/2.61 2-Undecanone 44.92/48.49 Tetradecanal 1.48/0.82 β-Pinene 9.95/8.67 β-Myrcene 17.68/18.36 (E)-3,7-Dimethyl-2,6-octadien-1-ol 1.55/2.44 Bornyl acetate 1.89/1.28 Geranyl acetate 2.65/0.75 Phytol 1.06/0.19 Nonterpene compounds 58.34/57.66 Aliphatic compounds 58.04/57.50 Terpenoids 41.66/42.34 Terpene hydrocarbons 31.95/35.79 Monoterpene hydrocarbons 30.74/35.27 Oxygenated terpenes 9.72/6.55 4-Tridecanone 0.55/1.78 α-Pinene 0.63/2.18 (1S)-(-)-β-Pinene 0.77/1.64 Limonene 0.37/1.35 β-Phellandrene 0.53/1.82	[92,93,94,149]
5.	*Thymus vulgaris* L. (*T. vulgaris*)	It is involved in antiinflammatory processes by: suppressing transcription factors of the induction of cytokine expression in T cells, the AP-1 and nuclear factors of activated T cells (NFAT) reducing secretion of TNF-, IL-1β, CRP, IL-8 and increasing IL-10 production affecting activity of elastase produced by activated neutrophils and inhibiting COX inhibiting NF-κB and mitogen-activated protein kinase (MAPK) signaling pathways affecting TNF and IL-1β secretion and production of IL-2 and IFN-γ, involved in T cell activation affecting synthesis of LPS-mediated nitric oxide	The composition of essential oil isolated from aerial parts of the plant grown in Nyons, France/Jablanicki, Serbia/Pomoravje District, Serbia/Richerenches, France: α-Pinene 0.47/0.21/1.75/1.32 Camphene 1.17/0.38/0.2/1.19 Sabinene -/0.05/2.03 Myrcene 0.09/0.44/4.0/1.59 α-Terpinene -/-/2.65/1.30 p-Cymene 0.09/0.18/1.0/20.07 Limonene 0.05/0.39/2.8/0.39 γ-Terpinene -/0.09/4.58/9.03 cis-Sabinene hydrate -/0.31/30.77/0.17 Linalool 76.15/7.15/7.89/5.00 Camphor 1.79/0.11/0.1/1.42 Borneol 0.40/1.00/0.28/1.50 Terpinen-4-ol 0.06/0.17/9.5/1.25 α-Terpineol 0.11/0.09/2.69/0.16 7-Methylenebicyclo[3.3.1]nonan-3-ol -/-/6.07/- Linalyl acetate 14.26/-/3.40/- Geraniol -/59.75/0.24/0.05 Geranial -/1.25/-/- Thymol -/0.42/-/47.06 Carvacrol 0.05/-/-/3.24 cis-p-Menthadienyl acetate -/0.19/4.75/- Geranyl acetate 0.06/16.72/0.48/- β-Caryophyllene 2.27/3.67/2.03/1.79 Geranyl propanoate -/1.26/-/-	[8,97,98,99,100,101,150]
6.	*Artemisia judaica* L. (*A. judaica*; Asteraceae; Compositae)	It exhibits antiinflamamtory activity by: reducing the TNF and promotes TGF-β1,IL-6, IL-8, leukotriene B4 (LTB4), PGE 2, and IL-1 downregulating 5-lipoxygenase (LOX) and COX	The composition of essential oil isolated from leafs of the plant grown in Saudi Arabia/Algeria/Egypt/Sinai, Egypt/Jordan/Libya: Piperitone -/66.17/49.1/29.9/3–15/30.2 Camphor -/-/34.5/31.4/0.3–16/- cis-Thujone 2.5/-/-/-/-/- thymol 3.5/-/-/-/-/- trans-sabinyl acetate 3.3/-/-/-/-/- carvacrol 3.5/-/-/-/-/- b-eudesmol 13.1 /-/-/-/-/- eudesma-4 (15), 7-dien-1-b-ol 3.5/-/-/-/-/- hexadecanoic acid 5.7/-/-/-/-/- ethyl cinnamate isomer -/6.11/-/-/11/3.8 spathulenol -/2.34/-/-/-/- E-longipinane -/2.55/-/-/-/- Borneol -/-/3.90/5.72/-/- Artemisia ketone -/-/-/-/9–24/- chrysanthenone -/-/-/-/4–31/- cis-Chrysanthenol -/-/-/-/-/9.1	[9,10,107,108,109,110]
7.	Patchouli (*Pogostemon cablin*, *P. cablin*)	Plays a role in antiinflammatory procesess and wound healing by: differentiating between M1 and M2 macrophage phenotypes changing the inflammatory milieu of ApcMin/+ mice, inhibit CD4+CD25+, while stimulating CD4+ and CD8+ cells in the spleen, blood, mesenteric lymph nodes (MLNs), and Peyer’s patches (PPs) of treated mice	The composition of essential oil isolated from leafs of the plant grown in Aceh Indinesia/India: Cis-alpha-Guaiene 1,44/14.6 Methanoazule 4,94/- Germacrene 2,3/-1 Bicycloheptane 3,87/- Patchouli alcohol 42.75/23.2 Delta-Guaiene 28.30/- Azulene 20.48/- Trans Caryophellene 11.84/- Seychellene 10.77/5.6 Nephtalene 8.02/- Cycloheptane 6.02/- Alpha Patchoulene 3,53/3.3 Caryophyllene 5.73/4.5 a-pinene -/0.2 b-pinene -/0.4 isoledene -/2.6 a-copaene -/1.7 b-patchoulene -/4.2 a-humulene -/0.7 selinene -/3.9	[111,151]
8.	*Echinacea purpurea* (*E. purpurea*) L. Moench (Asteraceae)	It supports the immune system by stimulation of monocytes and NK cells	The composition of essential oil isolated from the leaf/root of plant grown in South Africa: α-Pinene 2.8/3.7 β-Pinene 1.6/1.2 α-Phellandrene 6.9/6.6 π-Cymene 3.7/2.9 Limonene 2.3/1.7 Linalool 1.5/2.7 Camphor –/3.4 Borneol –/2.1 p-Cymen-8-ol –/1.2 Naphthalene 7.8/6.4 Estragole 2.2/3.3 Trans-Carveol 4.8/2.6 Cis-Carveol 1.0/1.0 Carvone 1.0/3.8 Peperitone 1.8/- α-Cubebene 1.4/0.9 Caryophyllene 4.5/4.0 γ-Muurolene 1.2/- Germacrene D 18.1/20.3 Caryophyllene oxide 11.3/12.2 Viridiflorol2.9/- Cedrol 7.2/10.5 Ledol –/3.1 α-Cadinol 9.1/5.9	[113,114,152]
9.	Cardamom (Elettaria cardamomum Maton, Zingiberaceae)	It is involved in antiinflammatory response by: reducing the presence of macrophages, monocytes, T lymphocytes in the colonic mucosa and lamina propia and decreasing secretion of nitric oxide, TNF, and IFN-γ in the intestinal tract suppressing secretion of Th1 cytokines including IFN-γ in murine splenocytes and TNF production by mouse peritoneal macrophages	The composition of essential oil isolated from the grains/fruit of the plant grown in Jordan/Mersin Turkey: 1,8-Cineole 8.82/25.6 Linalool 6.99/6.4 Terpinen-4-ol 1.83/2.8 Dihydrocarveol 6.06/ Geraniol 4.46/1.6 δ-Terpinyl acetate 55.99/40.7 Eugenol 2.31/ Z-Caryophyllene 3.82/ E-Nerolidol 3.07/- α-Pinene -/1.4 Sabinene -/2.1 Myrecene -/1.4 Limonene 1.1/- Linalyl acetate -/2.0	[125,153]
10.	Citrus sudachi (sudachi oil, Rutaceae)	suppresses properties of anti-gen-presentation-related molecules on dendritic cells in vitro suppresses antigen-specific T cell induction in vivo in mice immunized with OVA [127]. suppresses antigen-induced lymphocyte activation	The composition of essential oil from fruit of the plant grown in Kagoshima, Japan/Yamaguchi, Japan: α-Pinene 1.78/1.18 β-Myrcene 2.97/3.54 Limonene 60.30/71.34 β-Phellandrene 8.22/7.27 α-Terpinene 5.12/5.51 p-Cymene 6.08/3.56 γ-Elemene 1.93/- α-terpineol -/1.90 (E,E)-α-farnesene -/1.00	[127,154,155]
11.	*Euodia ruticarpa* (*E. ruticarpia*) (Rutaceae)	It exhibits anticancer propeties by: increasing the level of IL-1β, TNF-α, IL-6 and IL-10 secreted by macrophages affecting regulation of Th1 (IL-2) and Th2 (IL-10) cytokines production by splenocytes activating NK cells and B cells	The composition of essential oil from leaf /fruit: β-myrcene 31.03/38.14 β-phellandrene 18.38/25.89 trans-β-ocimene 3.35/- β-ocimene 3.21/17.82 (E)-4,8-dimethyl-1,3,7-nonatriene 6.17/- caryophyllene 2.21/- E-nerolidol 21.79/- γ-eudesmol 1.09/- β-eudesmol 1.52/- α-eudesmol 2.1/- α-pinene -/1.63 trans-β-ocimene -/12.29	[32,130,131,132,133,134,135,156]
12.	Eucalyptus plant (Myrtaceae)	Its topical application inhibits edema and enhances vascular permeability in IgE-mediated allergic dermatitis it may stimulate the immune function of the respiratory tract, improves phagocytic activity of macrophages and it can be used as an adjuvant in some infections and inflammatory diseases. It increases podosome density and augments colocalization at the podosomes of the actin core with the ring component vinculin and—takes part in macrophage motility and chemotaxis processes	The composition of essential oil from leaf of the plant Eucalyptus maculate/Eucalyptus globulus grown in Tanzania: α-Pinene 8.46/23.62 β-Myrcene 7.78/8.74 p-Cymene 10.10/10.00 Eucalyptol 54.29/51.62 γ-Terpinene 1.73/2.59 Terpinen-4-ol 0.04/2.74	[2,40,137,138,141,142,157]

**Table 2:** Influence of the most common essential oil constituents on the immune system (activity) and their chemical classification.

Lp.	Constituent	Activity	Chemical Classification	Literature
1	α-pinene	has no effect on human neutrophil Ca^2+^ influx is a very low inhibitor of neutrophil chemotaxis inhibits LPS-induced nitrite oxide secretion via reduction of inducible NOs mRNA and protein expressions in macrophages of rats reduces NOs mRNA and protein expressions in RAW 264.7 macrophages inhibits activation of IKK-β, NF-B in human mast cells blocks NF-κB and JNK activation in chondrocytes diminishes the production of IL-6, TNF-α, PGE2, NO production in LPS-stimulated peritoneal macrophages in mice diminishes COX-2 expression in LPS-stimulated peritoneal macrophages in mouse diminishes LPS-induced phosphorylation of MAPKs ERK and JNK in LPS-stimulated peritoneal macrophages in mouse affects IKK expression, p-IB level, NF-B translocation in LPS-stimulated peritoneal macrophages in mouse	Monoterpene hydrocarbons	[158,159,160,161,162,163]
2	*p*-cymen-8-ol	has no effect on human neutrophil Ca^2+^ influx does not affect neutrophil chemotaxis	Monoterpene alcohol	[158,160]
3	limonene,	has no effect on human neutrophil Ca^2+^ influx diminishes chemotaxis of neutrophils and leukocytes when combined with p38 mitogen-activated protein kinases (MAPK) inhibitor reduces leukocytes infiltration reduces levels of TNF-α, IL-1β, IL-6 in in LPS-stimulated RAW 264.7 macrophages reduces the expression of inducible NOs, COX-2 and production of PGE2 in LPS-stimulated RAW 264.7 macrophages reduces the activity of NF-κB in the rat kidney model inhibits mast cells activation and degranulation in the skin	Cyclic monoterpene	[158,164,165,166,167]
4	β-pinene	has no effect on human neutrophil Ca^2+^ influx, but (1*S*)-(−)-β-pinene stimulated neutrophil Ca^2+^ influx inhibits neutrophil migration inhibits the formation of 5-LOX (lipoxygenase) in 5-LOX assay	Monoterpene hydrocarbons	[158,168]
5	camphor	has no effect on human neutrophil Ca^2+^ influx inhibits production of IL-1β, IL-4, TNF-α in gingival tissues inhibits chemotaxis and leukocyte infiltration by diminishing the activity of myeloperoxidase enzyme (MPO) inhibits phorbol-12-myristate-13-acetate/ionomycin (PMA/I)-induced secretion of IL-6 and IL-8 by human gingival fibroblasts	Monoterpenoid	[110,169,170]
6	myrcene	has no effect on human neutrophil Ca^2+^ influx inhibits NO production in macrophages stimulated by LPS downregulates the production of NO induced by IL-1β in kidney tissues from ADX rats (after andrenalectomy) reduces NF-κB and inducible NOs expression induced by IL-1β in kidney tissues from ADX rats (after andrenalectomy) downregulates IL-6, IL-1β, IL-4, TNF-α COX-2 in kidney tissues from ADX rats (after andrenalectomy) increases the level of IFN-γ in kidney tissues from ADX rats (after andrenalectomy) upregulates IL-10 in kidney tissues from ADX rats (after andrenalectomy)	Monoterpene hydrocarbons	[158,165,171]
7	β- and α-phellandrene	do not inhibit human neutrophil chemotaxis reduce the total human leukocyte influx block NF-kB activity from LPS-stimulated macrophages inhibit neutrophil infiltration and mast cell degranulation reduce production of TNF-α, IL-6, COX-2, IL-1β by LPS-stimulated macrophages decrease the leukocyte migration via: ✓ blocking of TNF-α and IL-6 by resident cells ✓ changing expression of adhesion molecules in endothelial and polymorphonuclear cells inhibit inducible NOs, COX-2 gene transcription as well as IL-1 production by LPS-stimulated macrophages	Monoterpene hydrocarbons	[167,172,173,174,175,176]

8	spathulenol	inhibits neutrophil chemotaxis downregulates lymphocyte activation in a dose-dependent manner blocks IL-1β and IL-6 production by human lymphocyte inhibits human neutrophil function by affecting the activity of: ✓ MAPKAPK2 (MAP kinase-activated protein kinase 2) ✓ JNK3 (mitogen-activated protein kinase 10) ✓ Integrin α-L ✓ BMP2 (bone morphogenetic protein 2)	Oxygenated sesquiterpene	[172,177,178,179,180,181]
9	myristicin	inhibits human neutrophil [Ca^2+^] influx is hardly able to inhibit neutrophil migration	Phenylpropene	[158]
10	myrtenol	reduces human neutrophil migration reduces myeloperoxidase (MPO) activity in peritoneal fluid collected after carrageenan-induced peritonitis in the mice model reduces IL-1β production in peritonitis in the mice peritonitis model	Monoterpene alcohol	[158,182]
11	(+/−)-sabinene	stimulates human neutrophil Ca^2+^ influx blocks neutrophil migration blocks inducible NOs expression, in LPS-induced Raw 264.7 macrophages	Monoterpene hydrocarbons	[158,183,184]
12	γ-terpinene	stimulates human neutrophil Ca^2+^ influx blocks neutrophil migration inhibits expression of inducible NOs mRNA in LPS-induced Raw 264.7 macrophages	Monoterpene hydrocarbons	[158,163]
13	terpinen-4-ol	has no effect on human neutrophil Ca^2+^ influx inhibits production of TNF*α*, IL-1*β*, IL-6, IL-8, IL-10, PGE_2_ by human LPS- stimulated monocytes diminishes IL-1β, IL-6 and IL-10 production by human TLR4 and TLR2/TLR4-activated macrophages	Oxygenated monoterpene	[185,186,187]
14	terpinolene	has no effect on human neutrophil and FPR1-HL60 cells Ca^2+^ influx improves proliferation and migration of L929 fibroblast cells inhibits NO production by LPS-stimulated RAW 264.7 cells its pre-incubation with RAW 264.7 macrophage cells leads to suppression of superoxide anion generation in LPS-stimulated macrophages diminishes IL-6 and TNF-α secretion in LPS-stimulated RAW 264.7 macrophage cells inhibits NF-κB activation in human embryonic kidney (HEK) 293 cells	Monoterpene hydrocarbons	[158,160,188]
15	*p*-cymene	has no effect on human neutrophil Ca^2+^ influx blocks NO secretion via reduction of inducible NOs mRNA and protein expressions in RAW 264.7 cells inhibits IL-6 production in the mouse model of colitis forms 5-LOX (lipoxygenase) in 5-LOX assay	Monoterpene	[98,163,168,185,189]
16	nerolidol	inhibits agonist-induced activation of human neutrophils reduces CXCL1, IL-8, CCL2, mRNA expression in HT-29 cells reduces IL-6, IL-1β, TNF-α production by dextran sodium sulfate (DSS)-treated C57Bl6 mice reduces COX-2, inducible NOs proteins expression in the rat model of neuro-inflammation diminishes the influx of polymorphonuclear cells in carrageenan-induced peritonitis in mice	Oxygenated sesquiterpenes	[185,190,191]
17	myrtenol	has no effect on human neutrophil Ca^2+^ influx inhibits chemotaxis of human neutrophils reduces TNF-α, IL-1β, IL-6 levels in the peritoneal exudate of mice with carrageenan-induced peritonitis	Monoterpene alcohol	[158,182,192]
18	(±)-bornyl acetate	activates human neutrophils (via Ca^2+^ influx) inhibits human neutrophil migrations reduces IL-11 mRNA and protein expression in human chondrocytes acts antagonistically on IL-1β upregulation of matrix metalloproteinase (MMP) MMP-1 and MMP-13 in human chondrocytes diminishes the number of LPS-stimulated neutrophils and macrophages in murine bronchoalveolar lavage fluid blocks expression of IL-1β, TNF in human umbilical vein endothelial cells (HUVECs) and RAW 264.7 macrophages reduces LPS-induced activation of MAPK and NF-κB in acute lung injury and acute respiratory distress syndrome (ALI) model in mice diminishes LPS-induced secretion of TNF-α, IL-1β and IL-6 in the mouse model of acute lung injury and acute respiratory distress syndrome (ALI)	Bicyclic monoterpene	[185,193,194,195,196]
19	borneol	inhibits *f*MLF- and WKYMVM-stimulated neutrophils inhibits phorbol-12-myristate-13-acetate/ionomycin (PMA/I)-induced secretion of IL-6 and IL-8 by human gingival fibroblasts inhibits TNF-α, IL-1β, IL-6 and IL-8 secretion by LPS-stimulated monocytes block production of NF-ĸB, COX-2, LOX-5 in human rheumatoid disease	Oxygenated monoterpenes	[110,185,197,198].
20	geraniol	leads to prostate cancer cells autophagy via blocking Akt signaling and activation of AMPK signaling diminishes IL-6 and TNFα productions by BV-2 cells	Monoterpenoid	[169,199,200]
21	o-cymene	diminishes carrageenan induced TNF-α secretion in the mouse model of pleurisy affects carrageenan-induced migration of leukocytes, neutrophils in the mouse model of pleurisy reduces production of TNF-α, IL-1β and IL-6 in the mouse model of LPS-induced acute lung injury reduces the number of neutrophils and macrophages in the mouse model of LPS-induced acute lung injury reduces phosphorylation of JNK, ERK1/2, p38 MAPK in the mouse model of LPS-induced acute lung injury inhibits NF-κB kinases in the mouse model of LPS-induced acute lung injury	Monoterpene	[201,202]
22	1.8-cineole	reduces IL-4, IL-5, IL-10 production in nasal lavage fluids and the levels of IL-1β, IL-6, TNF-α and IFN-γ in lung tissues in influenza viral infection in mice improves IκBα proteins level, which leads to inhibition of the nuclear import of NFκB in the human cancer cell lines U373 and HeLa inhibits the NOD-like receptor pyrin domain-containing 3 (NLRP3) activity—regulator of IL-1β secretion and caspase-1 activity as well as diminishes NF-κB and p38 activity in murine alveolar macrophages affects M2 macrophages polarization by binding to peroxisome proliferator-activated receptor-γ (PPARγ) in the mice model of dextran sodium sulfate (DSS)-induced colitis has no effect on human neutrophil Ca^2+^ influx diminishes leukotriene B4 and prostaglandin E2 production by monocytes in the human model of asthma inhibits phorbol-12-myristate-13-acetate/ionomycin (PMA/I)- induced secretion of IL-6 and IL-8 by human gingival fibroblasts inhibits TNF-α, IL-1β, IL-6 and IL-8 secretion by LPS-stimulated monocytes	Monoterpene	[110,126,160,161,198,202,203,204,205,206,207]
23	linalool	promotes IF-γ, IL−13, IL-2, IL-21, IL-21R, IL-4, IL-6sR, TNF-α secretion in T-47D cells promotes CD40, IFN-γ, IL-12 p40, IL-13, IL-17F, IL-1β, IL-2, IL-21, IL-21R, IL-23p19, IL-4, IL-6sR and TNF-α production by human lymphocyte inhibits IL-6 and TNFα secretion by BV-2 cells has no effect on human neutrophil Ca^2+^ influx	Cyclic monoterpene	[185,201,208]
24	Cis thujanol	reduces expressions of IL-6 and TNFα mRNA in BV-2 cells promotes IL-6 production in BV-2 cells slightly reduces TNFα secretion in BV-2 cells inhibits phorbol-12-myristate-13-acetate/ionomycin (PMA/I)-induced secretion of IL-6 and IL-8 by human gingival fibroblasts	Monoterpenoid	[10,163,200]
25	b-caryophyllene	reduces neutrophils chemotaxis blocks IL1-β,TNF-α and IL-6 formation in murine macrophage J774A.1 cells	Sesquiterpene hydrocarbons	[168,177]
26	citronellal	decreases iNOS mRNA and COX-2 transcription in LPS-induced RAW 264.7 cells diminishes NO, PGE2 production in murine macrophage blocks carrageenan-induced neutrophil migration in rat carrageenin-induced pleurisy	Monoterpene	[167,209,210]
27	Alpha terpineol	reduces neutrophils migration inhibits IL-6 formation in epithelial buccal cells reduces production of IL-1β, Il-6 and TNF-α by LPS-induced human macrophages inhibits LPS-induced activation of NF-kB and ERK pathways but stimulates activation of p38 MAPK in human macrophages inhibits expression of IL-1β, IL-6, TNF-α, COX-2, inducible NOs in the mice model of bacterial vaginosis (BV) and vulvovaginal candidiasis (VVC) inhibits NF-κB activation in LPS-stimulated mice peritoneal macrophages downregulates the expressions of COX-2 and inducible NOs in LPS-stimulated mice peritoneal macrophages	Oxygenated monoterpenes	[174,176,187,197,211,212,213]
28	carvacrol	reduces IL-1β, PGE_2_ production and COX-2, IL-1β mRNA expression in the paw edema model enhances IL-10 mRNA expression in the paw edema model	Monoterpene	[214]
29	Thymoquinone	diminishes IL-1β, IL-6, TNF-α, IFN-γ and PGE2 production but increases IL-10 level in the rat model of arthritis diminishes inducible NOs mRNA and protein expressions in the rat model of arthritis and in RAW 264.7 macrophages inhibits COX2 expression and prostaglandins synthesis in mice with allergic airway inflammation inhibits 12-O-tetradecanoylphorbol-13-acetate (TPA)-stimulated COX-2 expression as well as NF-κB activation in the nude mouse skin model its supplementation in streptozotocin (STZ)-stimulated diabetic blocks COX-2 expression in pancreatic tissue in the rat model of diabetes when injected intraperitoneally, reduces the number of blood leukocytes and plasma IL-6 level in the murine model reduces IgE level in serum in the rodent model of airway allergic inflammation reduces TNF-α mRNA levels in the lungs in the rodent model of airway allergic inflammation reduces TGF-β1 mRNA and inducible NOs gene transcripts level in the rodent model of airway allergic inflammation	Monoterpene	[164,215,216,217,218,219,220,221,222]

Abbreviations: COX—Cyclooxygenase; IL—Interleukin; inducible NOs—Inducible Nitric Oxide Synthase; LOX—Lipoxygenase; LPS—Lipopolysaccharide; NF-kB—Nuclear Factor kappa β; NO—Nitric Oxide; PGE2—Prostaglandin E2; PLA2—Phospholipase A2; MAPKs—mitogen-activated protein kinases; ERK—extracellular signal-regulated kinases; IKK-β—IκB kinase subunit–β; NF-β—Nuclear Factor kappa β; MPO—myeloperoxidase enzyme; (PMA/I)—phorbol-12-myristate-13-acetate/ionomycin; MAPKAPK2—MAP kinase-activated protein kinase 2; JNK—c-Jun N-terminal kinase; BMP2—bone morphogenetic protein 2; DSS—dextran sodium sulfate; TLR—Toll-like receptor; MMP—metalloproteinase; ALI—acute respiratory distress syndrome; Akt—Protein kinase B; IκBα—inhibitors of NF-κB; NLRP3—NOD-like receptor pyrin domain-containing 3; PPARγ—peroxisome proliferator-activated receptor-γ; DSS—dextran sodium sulfate; TPA—12-O-tetradecanoylphorbol-13-acetate; STZ—streptozotocin; TGF-β1—Transforming growth factor B; BV—bacterial vaginosis; VVC—vulvovaginal candidiasis.be presented in Table 2.

## 5. Conclusions

Traditional medicine has used various plants to treat health problems for centuries. This review describes selected essential oils exhibiting immunostimulating properties. Although new synthetic drugs have been invented, methods supporting their treatment are still being sought, especially in the treatment of infectious diseases. The ubiquitous growing drug resistance of etiological factors of infections is becoming a serious therapeutic problem both in inpatient (hospital infections) and outpatient treatment. This problem can be solved by the application of essential oils, which possess many bioactive properties. Supporting the immune system is particularly important in the treatment of infectious diseases of various etiologies. This is of great importance, especially to avoid recurrences, and is also necessary in the treatment of chronic infectious problems such as skin and subcutaneous tissue infections, including difficult-to-heal wounds and ulcers. The active compounds of many essential oils, by modulating the immune response, have an inhibitory effect on the inflammatory process. It is known that components of essential oils generate a specific synergistic effect that cannot be generated by synthetic drugs. It is also possible that components of oils positively interact with recommended anti-inflammatory and antimicrobial drugs. Thus, there is a need to explore possible synergies in future research to implement essential oils and their active ingredients in medical practice.

## Data Availability

Not applicable.
